# Buoyancy-Driven Chemohydrodynamic Patterns in A + B → Oscillator Two-Layer Stratifications

**DOI:** 10.1021/acs.langmuir.2c02548

**Published:** 2023-01-09

**Authors:** M. A. Budroni, L. Lemaigre, D. M. Escala, A. De Wit

**Affiliations:** † Department of Chemical, Physical, Mathematical and Natural Sciences, University of Sassari, Via Vienna 2, 07100 Sassari, Italy; ‡ Université Libre de Bruxelles (ULB), Nonlinear Physical Chemistry Unit, Faculté des Sciences, CP231, 1050 Brussels, Belgium

## Abstract

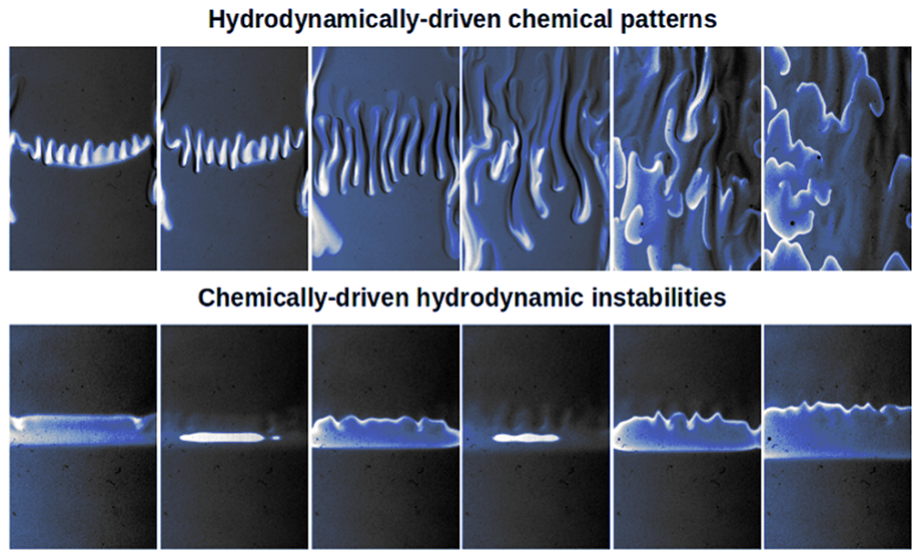

Chemohydrodynamic patterns due to the interplay of buoyancy-driven instabilities and reaction–diffusion patterns are studied experimentally in a vertical quasi-two-dimensional reactor in which two solutions A and B containing separate reactants of the oscillating Belousov–Zhabotinsky system are placed in contact along a horizontal contact line where excitable or oscillating dynamics can develop. Different types of buoyancy-driven instabilities are selectively induced in the reactive zone depending on the initial density jump between the two layers, controlled here by the bromate salt concentration. Starting from a less dense solution above a denser one, two possible differential diffusion instabilities are triggered depending on whether the fast diffusing sulfuric acid is in the upper or lower solution. Specifically, when the solution containing malonic acid and sulfuric acid is stratified above the one containing the slow-diffusing bromate salt, a diffusive layer convection (DLC) instability is observed with localized convective rolls around the interface. In that case, the reaction–diffusion wave patterns remain localized above the initial contact line, scarcely affected by the flow. If, on the contrary, sulfuric acid diffuses upward because it is initially dissolved in the lower layer, then a double-diffusion (DD) convective mode develops. This triggers fingers across the interface that mix the reactants such that oscillatory dynamics and rippled waves develop throughout the whole reactor. If the denser solution is put on top of the other one, then a fast developing Rayleigh–Taylor (RT) instability induces fast mixing of all reactants such that classical reaction–diffusion waves develop later on in the convectively mixed solutions.

## Introduction

How do chemical and hydrodynamic patterns interplay? This is one of the key questions at the heart of chemohydrodynamics, a growing research field at the intersection between chemistry and physics which studies the influence of reactive processes on the development of convective flows and *vice versa*.^1,2^ Advances in this area have recently born new insights into many fundamental and applied problems ranging from pattern formation and the origin of life^3−5^ to geologic carbon sequestration.^6^ Active chemohydrodynamic coupling is obtained when a chemical reaction, by changing *in situ* the properties of the fluid (such as density, viscosity, or surface tension), actively triggers or influences hydrodynamic instabilities. Here, we focus on buoyancy-driven hydrodynamic patterns in two-layer stratifications, when a solution of reactant A overlies a miscible solution containing reactant B and excitable or oscillatory regimes develop where A and B meet and react. Under nonreactive conditions, this kind of system can undergo a buoyancy-driven hydrodynamic instability not only if the density of solution A is greater than that of solution B, giving a typical Rayleigh–Taylor (RT) scenario, but also in initially stable stratifications if differential diffusion sets in when solute B initially dissolved in the lower denser solution diffuses faster than the top solute A, in which case a double-diffusive scenario (DD) can occur. *Vice versa*, a diffusive-layer convection instability (DLC) can develop if A diffuses faster than B.^7^ While under nonreactive conditions all of these scenarios develop symmetrically across the initial contact line between the two layers,^7−10^ a chemical reaction as simple as an A + B → C process can profoundly modify the symmetry of the fingered interface depending on the local density change induced by the reaction.^11−13^

A more complex scenario is encountered in the presence of nonlinear reactions able to sustain reaction–diffusion patterns such as autocatalytic fronts and waves. These can start convective flows due to localized compositional and temperature variations which, in turn, feed back with the spatiotemporal evolution of the chemical patterns. This active chemohydrodynamic loop has been shown to induce self-organized dynamics such as the acceleration and distortion of autocatalytic fronts,^14−16^ segmented chemical waves,^17^ oscillatory behaviors even in the absence of truly oscillatory chemical kinetics (i.e., in autocatalytic fronts and simple A + B → C systems),^18−22^ and transitions to chemical turbulence and chaos in spatially extended chemical oscillators.^23−27^ The potential of hydrodynamics in combination with oscillatory kinetics has also been introduced in neuromorphic engineering, chemical artificial intelligence, and chaos computing.^28,29^

In this context, the robust Belousov–Zhabotinsky (BZ) oscillator^30,31^ has been thoroughly used as a model system.^14,32^ The BZ mixture is a strongly acidic solution (10^–1^ M sulfuric acid is commonly used) containing bromate ions and an oxidizable organic substrate which, in the presence of a suitable one-electron redox catalyst (typically cerium(IV) salts or ferroin), can give rise to oscillations in the concentration of some of the reaction intermediates. From the kinetic viewpoint, the nonlinear mechanism can be minimally described as the alternation of an oxidation phase where an autocatalytic species forms and drives the system from the reduced to the oxidized state (thus ferroin, Fe(II) (red) → ferriin, Fe(III)(blue) if ferroin is used as a catalyst) and a second phase, the so-called “resetting of the chemical clock”, by which the reduced state (Fe(III) → Fe(II)) is restored through the oxidation of the organic substrate (typically malonic acid or 1,4-cyclohexanedione).^33,34^

If the BZ mixture is spatially distributed, then oxidation waves form and travel through the reactive medium, introducing potential sources of both buoyancy- and Marangoni-driven convection because the oxidized form of the catalyst can locally increase both the medium density and the surface tension.^14,17^ A rich variety of patterns due to this chemohydrodynamic coupling has been obtained in solution with an initial homogeneous distribution of the initial reactants.

**Figure 1:**
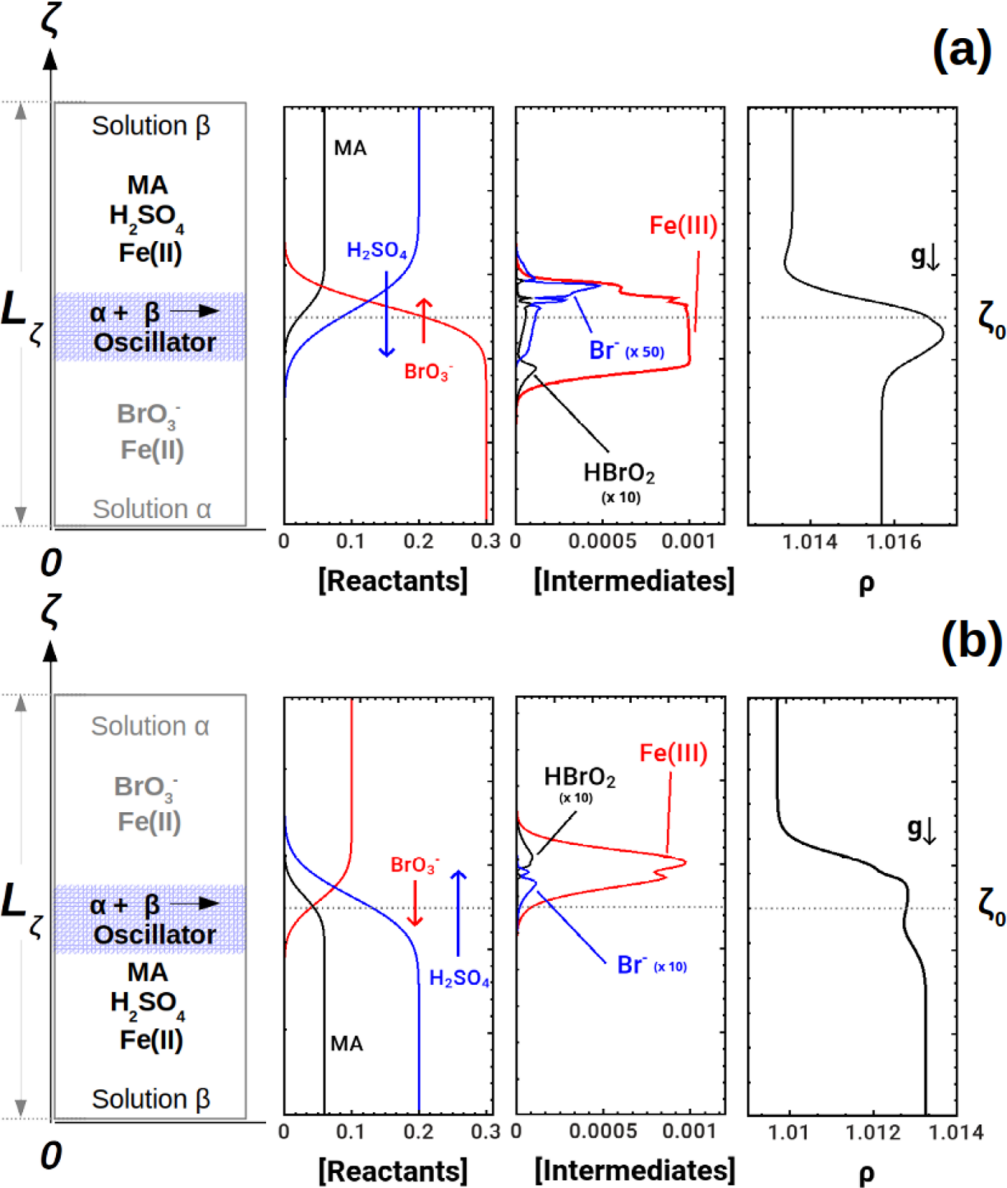
Sketch of the double-layer systems α + β → BZ where the acidic solution β is respectively on the top (upper panels, (a)) or on the bottom (lower panels, (b)). Panels (a) and (b) illustrate for both configurations the concentration profiles of the BZ reactants and intermediates forming in the mixing zone. Density profiles, shown on the right, can be reconstructed via eq 1 as a weighted combination of the species concentrations.

In this work, the cross influence of the BZ nonlinear kinetics and hydrodynamics is studied in a two-layer configuration typically adopted to study buoyancy-driven hydrodynamic instabilities, with the main reactants initially separated. To do so, we stratify in the gravity field solutions containing subparts of the BZ oscillator such that the reaction is initially localized across the mixing area of the two solutions, where the reactants meet by diffusion (sketches in Figure 1). This system features an extension of the well-known^35^ A + B → C systems coupled to buoyancy-driven flows^2,6,11,16^ to an A + B → oscillator problem.^36,37^

The first steps to deepen the dynamics of A + B → oscillator systems have been carried out by studying theoretically the reaction–diffusion (RD) properties of the Brusselator model sandwiched between cross-gradients of the main reactants.^37,38^ In parallel, combined experimental and numerical works have investigated the RD dynamics of localized pH oscillators,^39^ the chlorite–iodide–malonic acid (CIMA) reaction,^40^ and the BZ reaction spatially localized around the interface between two gels, one loaded with a solution containing bromate (solution α) and a second one with sulfuric acid and malonic acid (solution β) (both solutions containing ferroin with the same concentration).^36^ In the latter study, preparatory to this work, we showed how the resulting diffusion-limited patterns directly depend on the dynamical conditions (either excitable or oscillatory) across the initial contact area between the two layers.

By adding convection in two-layer A + B → oscillator configurations, we also numerically predicted with the Brusselator model the possibility of pulsating fingering and Turing spots ascending (or descending) in the gravitational field^41^ because of buoyancy effects induced by the concentration gradients. It was also found experimentally how the localized periodic forcing exerted by horizontal chemical waves generates transversely traveling fingering coupled to the wave dynamics.^42,43^

Here, we build on previous investigations of this class of systems to study experimentally how classical buoyancy-driven hydrodynamic instabilities such as DD, DLC, and RT modes can combine and influence RD structures developing around A + B → oscillator systems.^36^ Starting from buoyantly stable conditions, the initial separation of the reactants allows the activation of a DLC instability if the acidic solution β is stratified on top of a solution α of bromate because the protons coming from the dissociation of the sulfuric acid diffuse faster than bromate. The contact zone (where the reaction develops) undergoes a DD scenario in the reverse case where the acidic solution is layered on the bottom. RT modes will dominate these instabilities if the solution layered on top (either α or β) is denser than the bottom one. The active contribution of the chemical structures to the density distribution can further complicate these scenarios. We show how different chemohydrodynamic coupling schemes can be induced by controlling the type of buoyancy-driven instability expected under nonreactive conditions, the kinetic regime which is at play in the contact zone between the two layers, and the relative time scales characterizing the onset of the chemical and hydrodynamic instabilities.

## Methods

### Experimental Section

**Figure 2:**
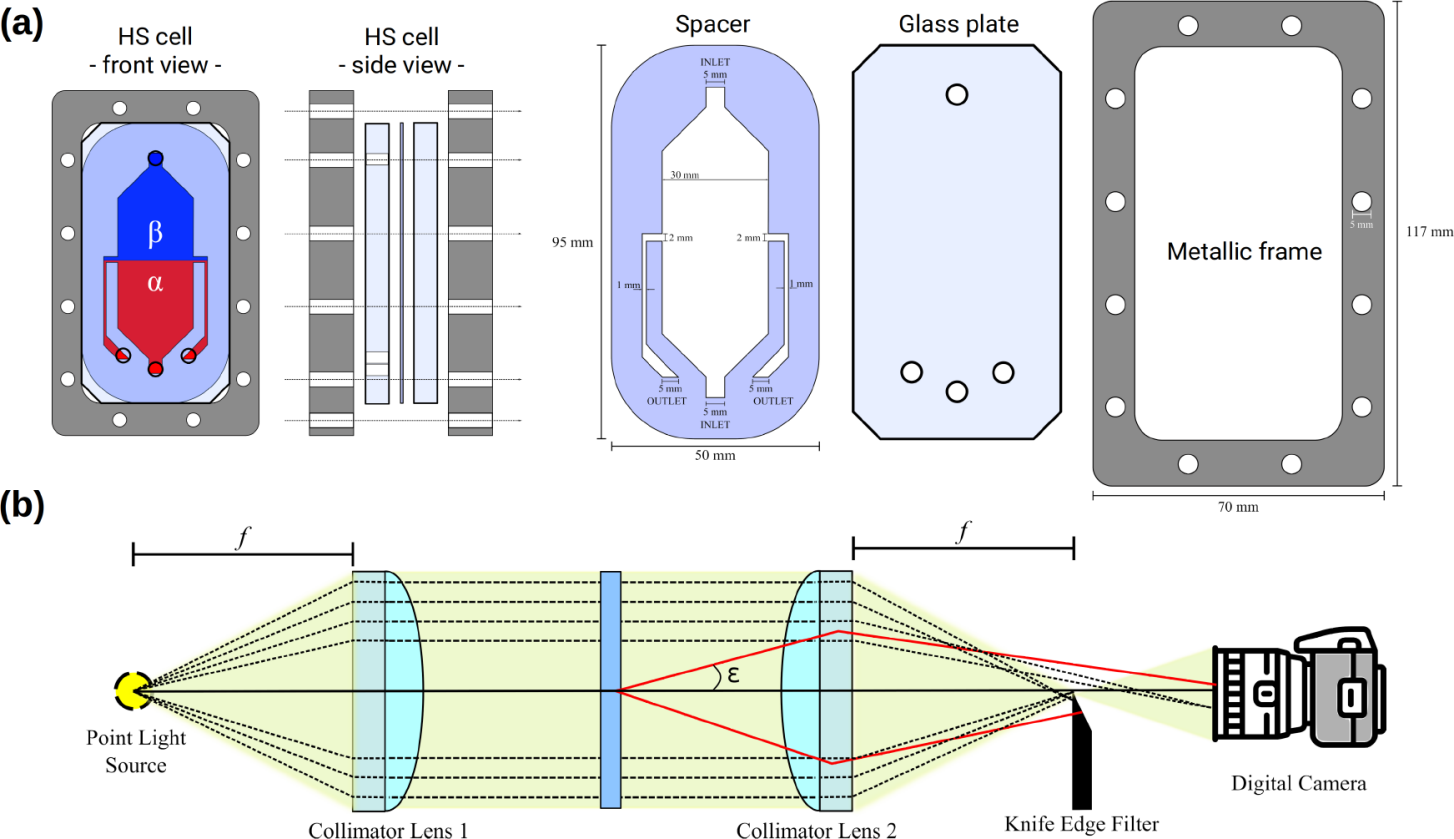
Sketch of the experimental setup. (a) Schematic of the Hele-Shaw (HS) cell and related exploded view showing the two glass plates (middle), a spacer (left), and a metal frame (right). (b) Basic features of a Schlieren optic setup: (from the left) point light source, collimator lens, the Hele-Shaw cell, collimator lens, cutoff, and collecting camera.

Experiments were performed by placing two aqueous solutions in contact, each containing a subpart of the BZ reactants, in a vertical Hele-Shaw (HS) cell, i.e., two glass plates separated by a thin gap^12^ (see Figure 2a). The two glass plates are made of borosilicate of optical quality (9.5 cm × 5 cm × 0.8 cm) separated by a thin spacer (0.5 mm) made of silicon rubber (Eriks, Belgium) which acts as a sealant to prevent leakage. The glasses and the spacer are held together by a metal frame. One of the glass plates contains four holes to allow the filling and emptying of the cell. Specific Teflon connectors are inserted into the holes and fixed to Teflon tubing by means of specific fittings (Bola, Germany). Inlet tubes are connected to two syringes containing the fluids to be injected. The outlet tubes are connected to a three-way stopcock that moved the exhaust liquids into a waste tank. The inner spacer is cut in the specific shape shown in Figure 2(a) to create a chamber for the two liquids as well as two exhaust channels on the sides.

To obtain the two-layer initial configuration shown in Figure 1, we employed the filling procedure of ref (44) and injected the two solutions manually with syringes. First, the cell was entirely filled with the bottom solution in order to replace the air contained in it. Then the top solution was injected with the outlets open in order to “push back” the interface between the two liquids to the midheight of the cell. Once a flat contact line between the two solutions was attained, outlets were closed and the experiment started.

The dynamics were visualized by the optical Schlieren technique, which allows to fluid motion to be followed by tracking refractive index variations,^45^ as illustrated in Figure 2b. Briefly, the light emitted by a point source is collimated by a lens and passes perpendicularly through the Hele-Shaw cell. It is refocused before hitting the objective of a camera. A cutoff, typically a simple razor blade, is placed at the focal point. Its role is to block most of the light which has not been deviated by the sample and reaches the focal point, as well as part of the light deflected by the sample. As a result, some of the deflected rays hit the camera objective and produce bright zones, while the rest is blocked by the cutoff and produces dark zones on the screen. Variations in the refractive index across the sample are thus converted to variations in intensity, and a grayscale image is obtained.

Following previous work,^36^ we separated the reactants of the BZ system into one solution containing sodium bromate and ferroin (solution α) and a second solution with malonic acid, sulfuric acid, and ferroin (solution β). The reference composition of both solutions was taken from ref (46) and then varied for our purposes. More precisely, the concentration of the main reactants placed on either side of the initial contact line, i.e., sodium bromate and malonic acid, was varied in order to explore different dynamical regimes of the BZ reaction but also various convective scenarios. In our parametric exploration, we kept the concentrations of sulfuric acid and ferroin constant. The concentration of the catalyst (ferroin) was kept the same in both solutions and was lower as compared to the reference recipe of ref (46) in order to make the solutions transparent enough for the concomitant visualization of the chemical patterns and fluid motions by the Schlieren technique. The concentrations of the solutions considered as well as their density are detailed in Table 1.

**Table 1:** Composition and Density of the Reactant Solutions Used in the Experiments^a^

fixed reactants	reactant varied	concentration (M)	density (g/cm^3^)	temperature (±0.1 °C)	solution number
Solution α					
ferroin	NaBrO_3_	0.100 ± 0.001	1.0099	21.5	α1
0.00100 M	NaBrO_3_	0.150 ± 0.003	1.0156	21.5	α2
±0.00002 M	NaBrO_3_	0.200 ± 0.002	1.0210	23.8	α3
	NaBrO_3_^b^	0.300 ± 0.004^b^	1.0331^b^	22.4^b^	α4^b^
ferroin 0 M	NaBrO_3_	0.100 ± 0.001	1.0097	21.5	α5
ferroin 0 M	NaBrO_3_	0.150 ± 0.003	1.0153	21.5	α6
Solution β					
	MA	0	1.0111	21.5	β1
	MA	0.0010 ± 0.0001	1.0112	21.5	β2
ferroin	MA^b^	0.0022 ± 0.0001^b^	1.0112^b^	21.5^b^	β3^b^
0.00100 M	MA	0.0050 ± 0.0002	1.0108	24.2	β4
+	MA	0.0100 ± 0.0005	1.0115	21.5	β 5
H_2_SO_4_	MA	0.100 ± 0.002	1.0140	24.8	β6
0.200 M	MA	0.400 ± 0.006	1.0250	23.5	β7
±0.005 M	MA	0.60 ± 0.02	1.0328	22.7	β8
	MA	0.80 ± 0.01	1.0401	22.6	β9
ferroin 0 M +	MA	0	1.0109	21.5	β10
H_2_SO_4_ 0.200 M	MA	0.0022 ± 0.0001	1.0110	21.5	β11

a MA stands for malonic acid. The densities are given with an error of (± 0.0005 g/cm^3^). The temperature at which the density measurement was performed is given in the fifth column. The solutions without ferroin are used for control experiments. The last column attributes a number to each solution in order to identify it in the text.

b Reference concentrations taken from the recipe proposed in ref (46).

The solutions to be stratified into the HS cell were prepared by mixing suitable amounts of stock solutions in volumetric flasks. All reactants were commercial grade and used without any further purification. The stock solutions of the reactants were prepared with distilled water and NaBrO_3_ (Sigma, puriss. p.a.), H_2_SO_4_ (Fluka, volumetric solution), and malonic acid (Merck). Ferroin was prepared by mixing FeSO_4_·7H_2_O (VWR, reag. Ph. Eur.) and 1,10-phenanthroline (Fluka, puriss. p.a.) in a 1:3 molar ratio and stirring the solution for several hours (overnight). All experiments were carried out at room temperature.

The diffusion coefficients of the reactants are given in Table 2. Based on these, it is possible to predict the type of convective instability which would take place if only two of the BZ reactants were placed in contact, without any chemical reaction for initially stable stratifications (bottom solution denser than the top one). This can guide the selection of “background” buoyancy-driven mechanisms to be activated in the system as well as the understanding of the convective patterns resulting from the interplay with chemical processes.

**Table 2:** Diffusion Coefficients and Density Expansion Coefficients of the Main BZ Species^48,49^^a^

Chemical species, I	diffusion coefficient (×10^–5^ cm^2^ s^–1^)	∂ρ/∂[*I*] (g cm^–3^ M^–1^)
NaBrO_3_	1.405	0.114
malonic acid	0.916	0.036
H_2_SO_4_	2.411	0.064
Br^–^	2.08	0.079
Fe(III)	n.a.	1.000

a The value for Fe(III) is assumed to be larger than that of the ferroin, Fe(II), for which we measured  g cm^–3^ M^–1^.

The snapshots of the experiments correspond to a real size of 2 cm × 3 cm, and a scale bar corresponding to a length of 1 cm is displayed. Moreover, the position of the initial contact line is indicated by a horizontal line which has the same width as the picture. The stratification of the solutions (α on top of β or the opposite) is also mentioned in the figures. The contrast of the snapshots was enhanced with free ImageJ software.^47^

### Density Profiles

To interpret the dynamics observed in the experiments, we reconstruct the density profiles along the gravitational axis, ζ, as this vertical spatial dependence of density is at the basis of the hydrodynamic instability.^7,50^ Dimensional density profiles are computed using the density state equation
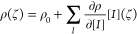
1using numerical concentration profiles [*I*](ζ) of the *I*th chemical species involved in the BZ system, weighted by related coefficients , giving the contribution of each species to the global density. (See the values specified in Table 2.)

The concentration profiles were computed as solutions of the dimensionless RD equations describing the species dynamics before the onset of convective instability:
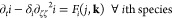
2The nonlinear reaction scheme is derived from the Epstein–Vanag model^51^ and described by the set of kinetic functions *F*_*i*_(*j*, **k**) depending on the concentration of the *j*th species and the set of kinetic parameters **k** as follows
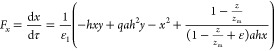
3
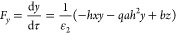
4
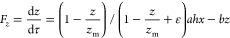
5

In this dimensionless model,^36^ variables *a*, *b*, *h*, *x*, *y*, and *z* are the dimensionless concentrations of the bromate, the organic substrate, the sulfuric acid, the autocatalytic intermediate HBrO_2_, the inhibitor Br^–^, and the oxidized form of the catalyst, ferriin, respectively. The kinetic parameters **k** = {ε, ε_1_, ε_2_, *q*, *z*_m_} are functions of the rate constants and the dimensional concentration of the initial reactants (*A*_0_ = bromate, *B*_0_ = malonic acid, *H*_0_ = sulfuric acid and *C*_0_ = ferroin) according to *q* = 2*k*_2_*k*_3_/(*k*_1_*k*_4_), ε_1_ = *k*_2_*H*_0_/*k*_4_, ε = *c*_min_/*C*_0_ (with ), and *z*_m_ = *Z*_0_/*C*_0_. (See the rate constants values reported in ref (36).)

Following the pool chemical approximation,^34^ we add *F*_a_ = *F*_b_ = *F*_h_ = 0 to the equation set (eqs 3–5), assuming that the spatiotemporal concentration of the reactants evolves according to eq 2 solely due to diffusion. In other words, we neglect the depletion of the reactants as they are slowly consumed as compared to the time scale of the oscillatory intermediate dynamics, which is an assumption that is particularly reliable for the BZ reaction.

The resulting dimensional concentrations correspond to [A] = *A*_0_*a*, [B] = *B*_0_*b*, and [H] = *H*_0_*h* for the reactants and to [X] = *X*_0_*x*, [Y] = *Y*_0_*y*, and [Z] = *Z*_0_*z* for the intermediates with the concentration scales {*X*_0_ = *k*_4_*A*_0_*H*_0_/2*k*_3_, *Y*_0_ = *k*_4_*A*_0_/*k*_1_, and *Z*_0_ = (*k*_4_*A*_0_*H*_0_)^2^/*k*_3_*k*_6_*B*_0_}.

δ_*i*_ is the ratio of the diffusivity of the *i*th species to that of the bromate taken as reference. (See the values specified in Table 2.) The bromate diffusivity is also used to define the spatial scale  and thus the dimensionless space coordinate ζ = *Z̅*/*L*_0_, where *Z̅* represents the corresponding dimensional vertical spatial coordinate and *t*_0_ = 1/(*k*_6_*B*_0_) is the reaction time scale.^36^

The system (eq 2) is solved by using the Crank–Nicolson^52^ method on a one-dimensional spatial domain (a vertical cut of the double-layer stratification sketched in Figure 1) of dimensionless length *L*_ζ_ = 100, discretized over a grid of 200 points (i.e., spatial mesh *h*_ζ_ = 0.5), using an initial distribution of reactants as in experiments and no-flux boundary conditions at the boundaries of the spatial domain. Simulations are run using the integration time step *h*_τ_ = 1 × 10 ^–4^, which was tested to avoid convergence problems due to the stiffness of the differential equations (eqs 3–5). Solving eqs 2–5 gives the profiles of concentrations of all species in the BZ system, which then allows the reconstruction of the density profiles according to eq 1. Typical examples of the various profiles are given in Figure 1.

### Parameter Space

We carried out the exploration of chemohydrodynamic scenarios of a localized BZ reaction in a vertical setup by considering the following approach. Two different initial configurations of the double-layer system are used to select the buoyancy-driven hydrodynamic instability potentially at play: either solution α over β or *vice versa*. For both configurations, we varied the concentration of the bromate in solution α in the range [0.10, 0.30] M, keeping the malonic acid concentration constant, [MA] = 0.02 M. This allowed the tuning of the density difference between solutions α and β without significantly affecting the excitable dynamical regime at the initial contact line between the solutions. Analogously, we varied the concentration of the malonic acid in the range of [0.001, 0.800] M, keeping [BrO_3_^–^] = 0.3 M. This not only modulates the density difference between the stratified solutions but also controls to a wider extent the excitability^17^ of the BZ system, defined as the ratio . Varying [MA] for a fixed value of [BrO_3_^–^] then allows a transition to be induced from the excitable to the oscillatory regime around ζ_0_.^36^

**Figure 3:**
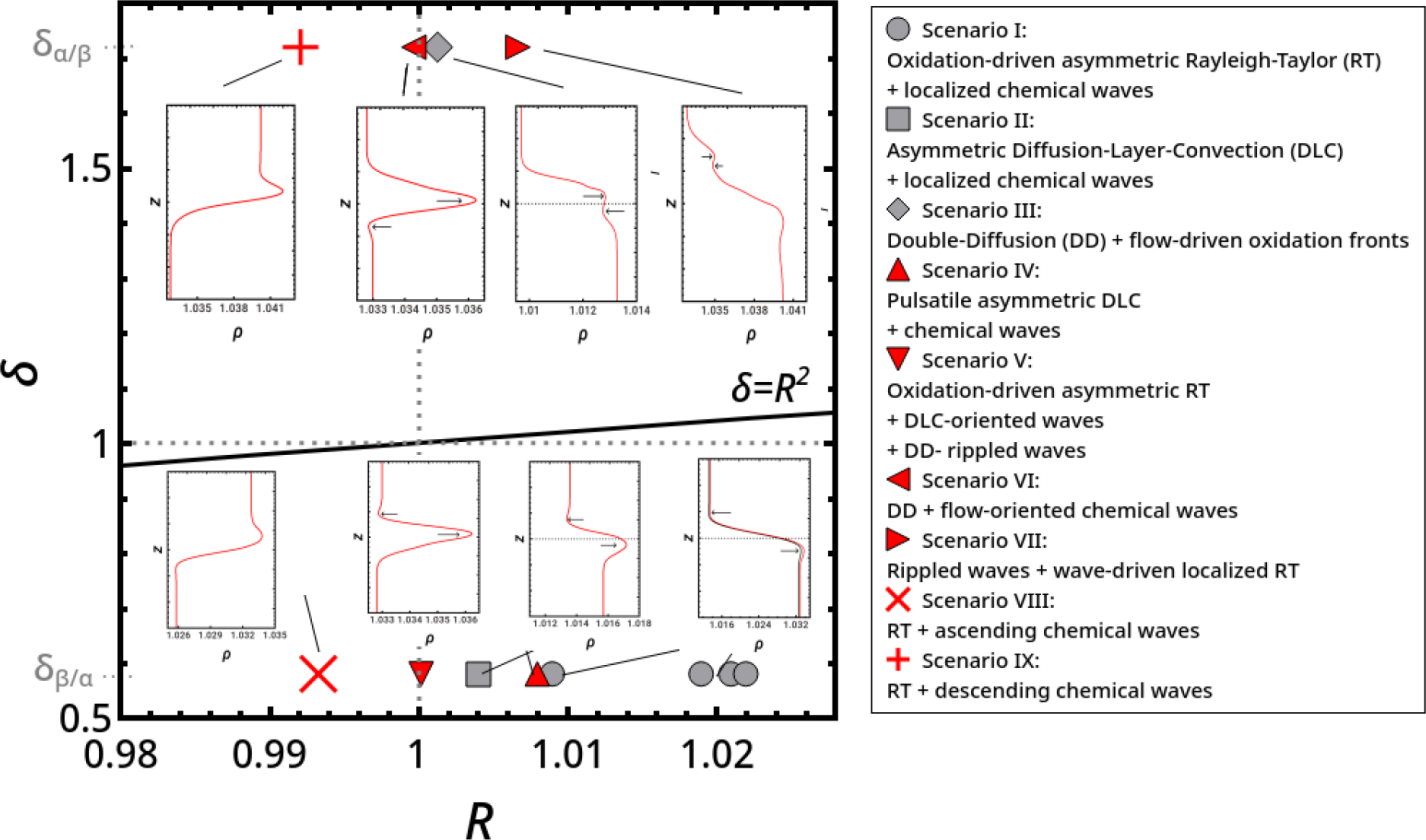
Classification of the main chemohydrodynamic scenarios in the parameter space spanned by the density ratio between the bottom and the top solution, *R* = ρ^bottom^/ρ^top^, and the diffusivity ratio, δ = *D*^bottom^/*D*^top^, defined using the sulfuric acid and bromate diffusivities. δ_α/β_ and δ_β/α_ identify the two diffusivity ratios characterizing the two reactant distributions considered. Different scenarios are accompanied by representative density profiles along the gravitational axis which can explain the observed dynamics. Scenarios obtained under excitable conditions across ζ_0_ are identified by gray symbols, and those corresponding to oscillatory regimes are in red.

Following the approach of ref (7), we classified the resulting chemohydrodynamic scenarios in the parameter space spanned by the density ratio between the bottom and the top solutions, *R* = ρ^bottom^/ρ^top^, and the diffusivity ratio δ = *D*^bottom^/*D*^top^, as shown in Figure 3. This gives an overview of the potential buoyancy-driven instabilities accessible to the system under nonreactive conditions and how these convective modes interplay with RD patterns. In such a parameter space, the system can undergo RT-like instabilities when *R* < 1, i.e., when ρ^top^ > ρ^bottom^. DLC and DD scenarios are located in the *R* > 1 region where δ < 1 and δ > *R*^2^, respectively. As explained in the Introduction, in our system, differential diffusion is essentially triggered by the different diffusivities of sulfuric acid and bromate, with MA playing a negligible role in the range of concentration considered. When solution α is on top, δ_α/β_ ≈ *D*^BrO_3_^–^^/*D*^H^+^^ ≈ 0.58 < 1. In that case, starting from a stable stratification (*R* > 1), differential diffusion sustains a DLC scenario. In the reverse configuration δ_β/α_ ≈ *D*^H^+^^/*D*^BrO_3_^–^^ ≈ 1.72 > 1 (*R* > 1), a DD instability is activated. In the parameter space of Figure 3, our experiments are thus located along two horizontal lines corresponding to the constant values of δ = 0.58 and 1.72. For each of them, *R* is varied by changing the reactant concentrations as described above. For different values of *R*, we show in Figure 3 representative density profiles along the gravitational axis reconstructed numerically thanks to eqs 1 and 3–5, taking into account the contribution of the localized reaction. The main scenarios described in the following text can be interpreted in terms of the morphology of these profiles. In Figure 3, the dynamics obtained under excitable conditions at ζ_0_ are identified by gray symbols while those corresponding to oscillatory regimes are in red.

## Results and Discussion

### Chemohydrodynamic Patterns in the Excitable Regime

The RD dynamics developing in gels on sides α and β when the BZ system is locally excitable around the contact line ζ_0_ were thoroughly analyzed in our previous work.^36^

Briefly, low values of the organic substrate (MA) concentration favor the formation of an oxidized front which propagates toward the side containing the bromate (solution α), and from this oxidized area, waves form and travel toward the side containing the organic substrate (i.e., solution β).

Let us describe what happens when convection comes into play in this excitable regime by analyzing the dynamics in vertical HS cells, starting from an initially buoyantly stable situation of a less dense solution above a denser one.

#### DLC Contributions

We start with stratifications of a less dense solution β over a denser solution α (Figure 1a) such that the fast diffusing acid is in the upper layer and DLC modes are expected (half line *R* > 1 and δ_β/α_ = 0.58 in Figure 2).

**Figure 4:**
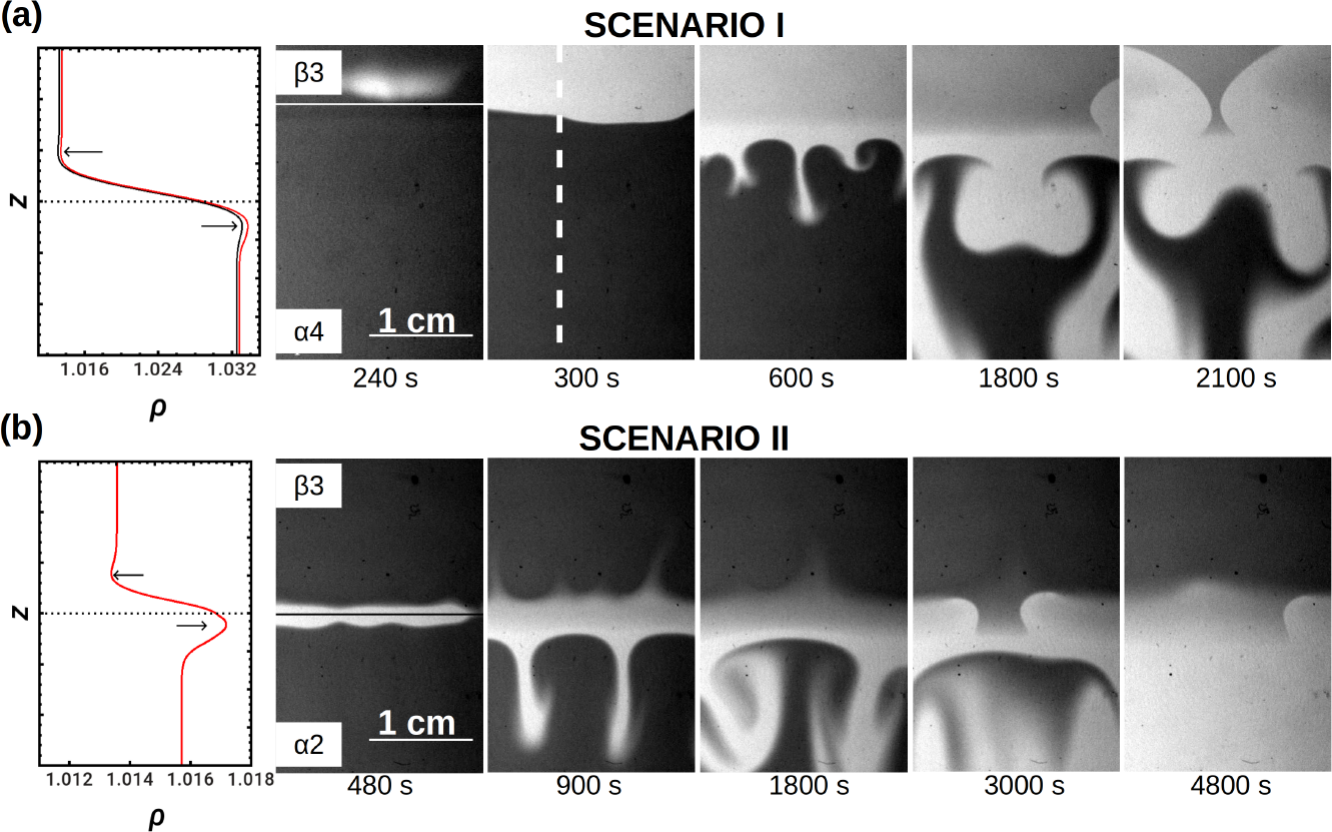
(a) Chemohydrodynamic scenario I develops when solution β3 ([MA] = 2.2 × 10^–3^ M) overlies the denser solution α4 ([BrO_3_^–^] = 0.3 M). Fingering in the bottom layer is due to the local accumulation of fast-diffusing sulfuric acid below the initial contact line and to the autocatalytic transformation of ferroin into denser ferriin. The contribution of the reaction is shown by comparing the density profile resulting from simple species diffusion (in black) and in the presence of the reaction (in red). (b) Scenario II: combination of ascending and sinking convective patterns obtained when solution β3 is put on top of solution α2 ([BrO_3_^–^] = 0.15 M). Diffusive layer convection sets in due to differential diffusion between the counter diffusing bromate salt and sulfuric acid. In both scenarios, chemical waves forming in the top layer remain localized. In each line, the first panel shows the reconstructed density profiles. The next panels present snapshots of the cell at successive times. Real size = 2 cm × 3 cm. The horizontal lines in the first snapshots of the dynamics indicate the position of the initial contact line while the vertical dashed line in the second snapshot indicates where space–time plots are built.

Scenario I. The typical spatiotemporal evolution characterizing experiments carried out with the reference concentrations (β3 and α4) is shown in Figure 4a. In order to start from a buoyantly stable configuration, the denser solution α4 is put below solution β_3_. Initially, both solutions are red (dark gray in the pictures). After a few minutes, a blue oxidized area develops (light gray in the snapshots) at the initial contact line, ζ_0_. Convective fingers emerge from this zone and sink, causing mixing in the bottom layer. After half an hour, waves nucleate at the lateral sides of the reactor, just above ζ_0_, and travel laterally toward the center of the HS cell where they collide and annihilate. These waves continue to travel long after the bottom of the reactor is mixed by convection and completely oxidized (light gray in the snapshots). Similar behavior was observed when the concentration of sodium bromate was slightly decreased, i.e., for [NaBrO_3_] = 0.2 M (solution α3).

**Figure 5:**
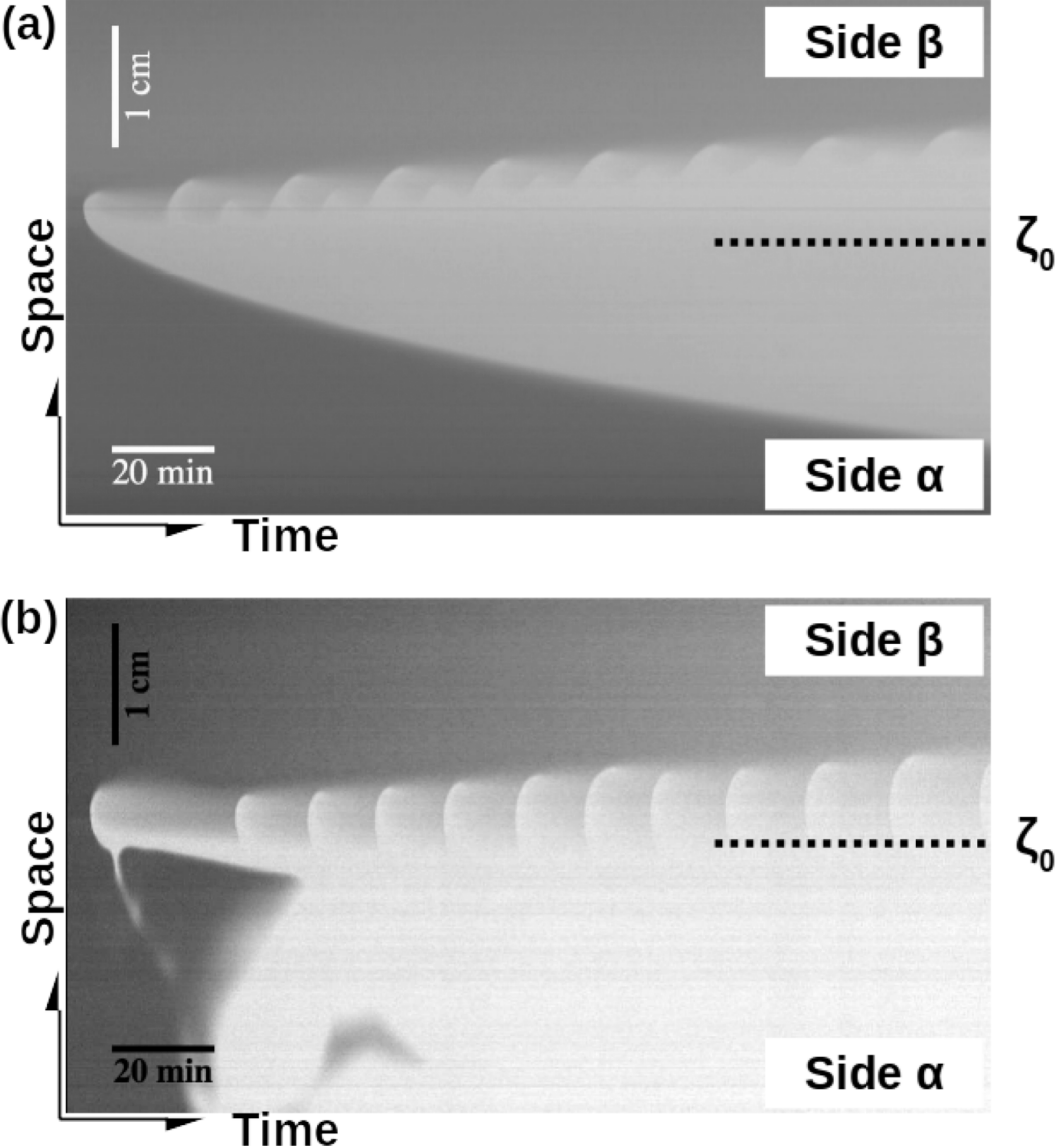
Space–time maps of the dynamics of the BZ reaction in the double-layer configuration in the (a) absence^36^ and (b) presence of buoyancy-driven flows when the system is locally excitable in the solution contact area (real dimensions = 4.2 cm × 180 min). (b) Space–time plot describes the experiment shown in Figure 4a along the vertical dashed line shown in the second snapshot.

Figure 5 compares the space–time plots obtained both in the (a) absence^36^ and (b) presence of convective motions, by stacking as a function of time a vertical section of the dynamics throughout the two layers. (See the dashed line in the second snapshot of Figure 4a.) The patterns observed in both cases are very similar, except that, in the presence of convection, waves nucleate preferentially at the side of the HS cell instead of along the whole extent of the contact line as in the RD case. With convection, the autocatalytic oxidation front traveling downward is now deformed into sinking fingers, and hence the lower side α is oxidized more quickly due to the gravitational currents. The hydrodynamic fingering can be attributed to two contributions. One is the accumulation of sulfuric acid below the initial contact line due to its higher diffusivity as compared to the counter-diffusiving bromate. For this configuration, a DLC scenario is expected under nonreactive conditions, with symmetric density depletion and accumulation zones, just above and below ζ_0_, respectively.^7,8^ However, there is a second additional contribution of the reaction which oxidizes *in situ* ferroin into denser ferriin.^15^ This enhances the local density maximum below ζ_0_ while the depletion area above ζ_0_ becomes negligible. As a consequence, an asymmetric density profile as shown in Figure 4a develops, with the local maximum in density below the contact line triggering the downward sinking fingers.

The onset of the convective motions is not directly related to the excitable nature of the reaction as confirmed by experiments performed in the absence of the organic substrate which is needed to observe waves (i.e., using solution β1 on top of α4). Hydrodynamic oxidized fingers still develop while the waves are no longer observed. We thus see that in the absence of gels, buoyancy-driven motions due to the local production of denser ferriin profoundly affects the reaction–diffusion patterning of the system.

Scenario II. If we decrease the concentration of the bromate salt in the bottom layer (solution α2, namely, [NaBrO_3_] = 0.15 M), then we decrease the density ratio between the two stratified solutions, i.e., decrease *R* (Figure 3), and additional features can then be observed (see Figure 4b). While fingering still occurs below the initial contact line, due to the maximum in density induced by the downward fast diffusion of acid and the local production of denser ferriin, additional convective patterns develop upward at a smaller growth rate. These plumes arise thanks to the minimum in density triggered by the depletion in acid (DLC mechanism) and entrain the oxidized solution from the reaction zone upward. To validate this interpretation, we performed nonreactive control experiments with no ferroin or malonic acid (thus placing a 0.2 M sulfuric acid solution on top of 0.15 M NaBrO_3_), indeed obtaining symmetric, purely diffusive DLC dynamics,^7−9^ as expected. The absence of the upward moving convective patterns in systems with larger [NaBrO_3_] (as in scenario I) is explained by the larger density jump between the two layered solutions occurring in that case, which stabilizes the interface. Indeed, in the previous cases (Figure 4a), *Δρ* has an order of magnitude of 10^–2^ g/cm^3^. On the contrary, for smaller [NaBrO_3_], *Δρ* is smaller (roughly 10^–3^g/cm^3^) and hence the density depletion zone above ζ_0_ is now not negligible any longer, as seen in Figure 4b.

The period of the waves and their induction time, measured from the space–time plots, did not show any clear dependence on the sodium bromate concentration in the range explored.

#### DD Contribution

**Figure 6:**
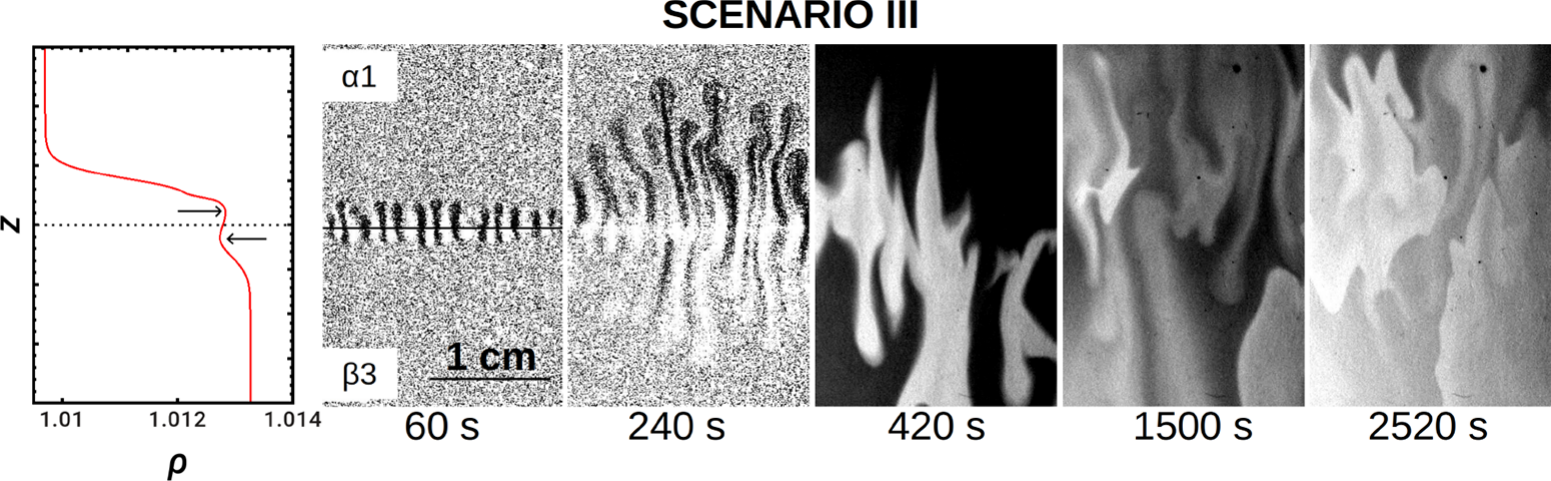
Scenario III: chemohydrodynamic patterns controlled by a double-diffusive (DD) instability quickly developing when the top solution α1 (with [NaBrO_3_] = 0.1 M) is put on top of a denser solution β3 ([MA] = 2.2 × 10^–3^ M). The first snapshots are made binary to obtain a better contrast. Real size = 2 cm × 3 cm. The horizontal line in the first snapshot of the dynamics indicates the position of the initial contact line.

In order to analyze the interaction of RD patterns with DD modes, we reverse the stratification such that the fast-diffusing acid is now in the lower layer (solution α over solution β as in Figure 1b). We then obtain scenario III. To make the upper solution α less dense than solution β, we further decreased the concentration of NaBrO_3_ to [NaBrO_3_] = 0.1 M (solution α1) and stratified this solution above the denser solution β3 (see Figure 6). In the parameter space of Figure 2, we are now in the region of *R* > 1, δ_α/β_ = 1.72. The equivalent nonreactive situation is obtained in the absence of ferroin and malonic acid, i.e., putting a solution of 0.1 M NaBrO_3_ (α5) on top of 0.2 M H_2_SO_4_ (β10).

In the nonreactive case, symmetric double-diffusive fingers deform the interface and extend vertically in the course of time.^8,9^ With the reaction at play, the interface also deforms rapidly in less than a minute into symmetric rising and sinking fingers before any oxidation process is visible (Figure 6). Later on, the oxidation process occurs within the convective motions and switches the fingered pattern on. Eventually, waves nucleate at random spots in the Hele-Shaw cell and propagate, distorted by the residual flow, in different directions instead of remaining localized along the initial contact line. The resulting chemical turbulence may be parallel to that in other experiments and to numerical studies where the onset of convection combined with BZ traveling waves was shown to be responsible for chemical spatiotemporal chaos.^23,25,41,53^

#### Difference between DLC and DD Contributions in the Excitable Regime

The comparison between Figures 4 and 6 allows us to draw the first conclusions about the mutual influence of chemical and hydrodynamic patterns in this excitable system. Switching from configuration β/α (DLC, scenarios I and II, Figure 4) to α/β (DD, scenario III, Figure 6) corresponds to an inversion of the characteristic time scales of the two key processes at play, namely, the autocatalytic ferroin oxidation and the nonreactive convective instability. In the first DLC cases, the hydrodynamic instability develops on a slow time scale such that the density changes induced by the chemical reaction have time to actively impact the hydrodynamics. On the contrary, in the more rapid DD case, the oxidation front is slaved to convection.

In both cases, waves nucleate at later times and their induction period plays a small role here. The effect of the intrinsic properties of the convective modes cannot be fully excluded though. The characteristic topology of DLC-type scenarios preserves the initial contact line of two stratified solutions, consisting of two independent separated convective zones. Hence, as seen in scenarios I and II (Figure 4), the upper layer is essentially governed by diffusion and reaction–diffusion waves localized within this diffusive layer maintain properties similar to those observed in the absence of convection.^36^ On the contrary, DD fingers cross the initial contact line and cause stirring along the whole spatial domain, hence waves cannot remain localized any longer (Figure 6).

### Chemohydrodynamic Patterns in the Oscillatory Regime

**Figure 7:**
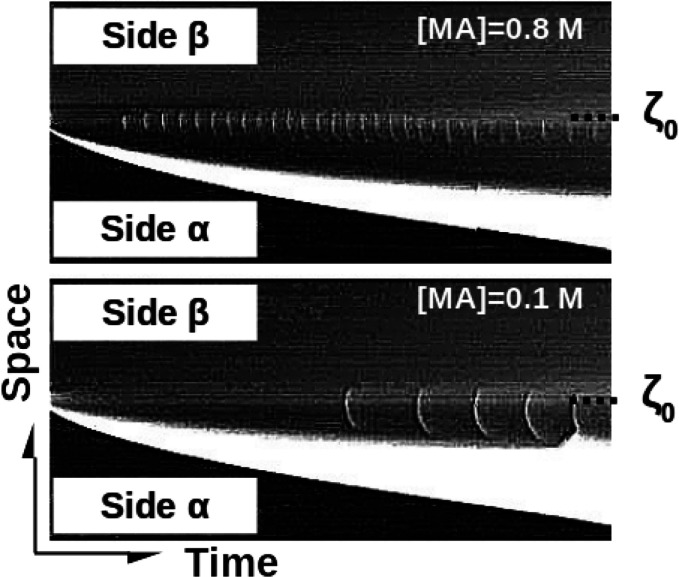
Typical space–time maps (dimensions 5 cm × 220 min) of the dynamics of the BZ reaction in the double-layer system in a gel, without buoyancy-driven flows when the system is locally oscillatory at the initial contact line between the two solutions (see ref (36)). Decreasing the concentration of malonic acid increases the period of the waves developing in the contact area.

We now study the effect of the transition from the excitable to the oscillatory regime at ζ_0_ by increasing the concentration of malonic acid and keeping the concentration of NaBrO_3_ fixed at 0.3 M in solutions α. From benchmark studies of the RD properties of these low excitability conditions,^36^ we point out how waves nucleate from the leading oxidized front in solution α as observed in previous excitable systems. This wave train presents a longer induction period and coexists with solitary pulsations developing in layer β where the medium is locally oscillatory. The bifurcation toward this second wave train can be observed for [MA] ≥ 0.1 M (see ref (36)) and is also visible around ζ_0_ in both space–time plots of Figure 7.

#### DLC Contribution

Let us look again at the effect of DLC modes by starting with a stratification of a less dense solution β over a denser solution α such that *R* > 1 and δ_β/α_ = 0.58. The equivalent points in the parameter space are shown in red in Figure 2.

Scenario IV. In the range of concentrations [MA] = [10^–3^, 10^–1^] M, the dynamics obtained are qualitatively similar to that shown in Figure 4a, with the oxidized front forming at the reaction interface and sinking as a fingered pattern into the underlying solution containing fresh reactants. Similarly, waves develop and are localized near the initial contact line, but since increasing the malonic acid concentration decreases the medium excitability, the morphology changes (waves are thinner) and the related wavelength increases.

**Figure 8:**
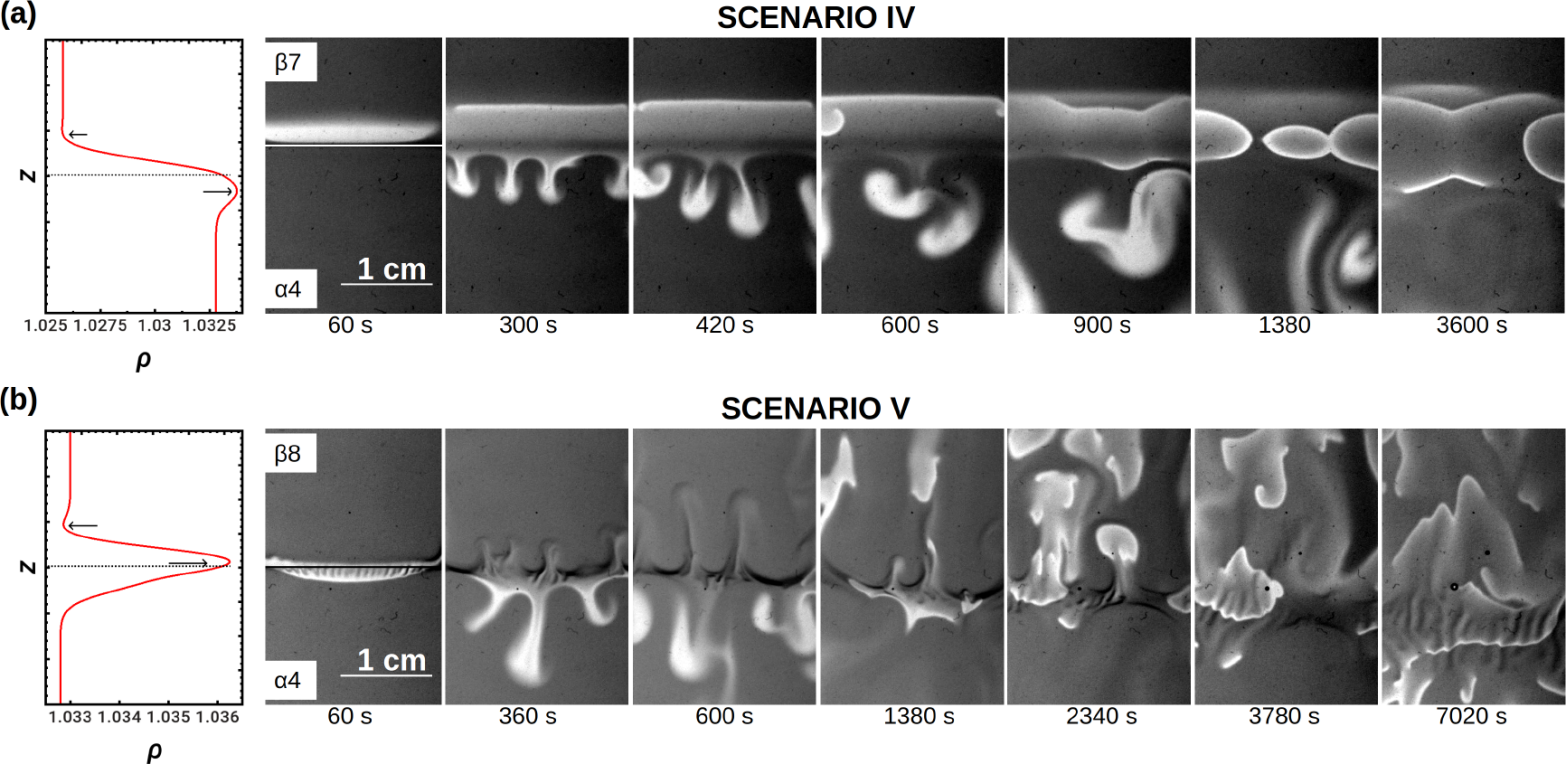
(a) Scenario IV: transient periodic fingering induced by the oscillatory kinetics in the reactive zone. Solution β7 is put on top of solution α4. (b) Scenario V showing three different convective instabilities concurrently at play and interacting with chemical waves in the isopycnal case. The dynamics are obtained with solution β8 ([MA] = 0.6 M) layered on top of α4. Waves no longer remain localized along the initial contact zone. Real size = 2 cm × 3 cm. The horizontal lines in the first snapshots of the dynamics indicate the position of the initial contact line.

When [MA] ≥ 0.1 M, a switch to a local oscillatory regime occurs and new reaction–diffusion–convection dynamics are obtained. In particular, at [MA] = 0.4 M (see Figure 8a), the planar oxidized front forming at ζ_0_ still deforms into fingers because of the increasing concentration of denser ferriin in the reaction zone. Nevertheless, owing to the oscillatory kinetics, the system can locally recover the reduced state producing periodic planar fronts moving toward the top side β, while convective fingers in the bottom layer grow and then detach from the main oxidized front as the less dense reduced form of the catalyst is restored. This scenario appears to be consistent with pulsatile fingering observed in our former numerical exploration with the Brusselator model.^41^ Interestingly, the return to the reduced state of the fingers tail does not affect the growth rate of these convective structures, and no significant difference was in general found when varying the concentration of the organic substrate in the range [MA] = [0, 0.4] M. The convective stirring of the bottom layer eventually leads to a bulk return to the initial reduced state instead of remaining uniformly blue as was the case for excitable scenarios I and II with a lower concentration of MA.

The morphology of this DLC-type scenario preserves the regular RD-like behavior of the waves that ultimately develop in the top layer as observed in scenarios I and II. Indeed, all these scenarios are characterized by a similar morphology of the density distribution along the gravitational axis, with a diffusion layer controlling the dynamics above ζ_0_ (See the first panels of Figure 8(a,b).)

Scenario V. With further increases in [MA], the initial density difference between solutions α and β can be progressively decreased close to the limiting case where the top and bottom layers have equal densities, i.e., isopycnal conditions, met for [MA] = 0.6 M (see Figure 8b). Here we can observe the concurrent interplay of three different hydrodynamic instabilities. As stated previously, the autocatalytic oxidation front produces sinking fingers due to denser ferriin. Moreover, the density jump between the top and bottom layers is small enough to allow the DLC instability due to the differential diffusion between NaBrO_3_ and H_2_SO_4_ to trigger large convective plumes rising in the upper solution. The sinking DLC convective plumes are, however, overwhelmed by the larger Rayleigh–Taylor fingers produced by the oxidation reaction. Finally, an additional instability develops at the interface with a smaller wavelength than for the two other patterns. The fingers are initially straight and regularly spaced, but their growth is later modulated by the DLC instability as they develop mainly at the base of DLC plumes (see the snapshot of Figure 8b at 300 s). At later times (after half an hour), these fingers elongate and deform while oxidation waves propagate through them. The onset of this additional fingering can be attributed to a DD mechanism involving the bromate salt underlying the less dense and slower-diffusing malonic acid: *D*_MA_ = 0.916 × 10^–5^ cm^2^/s < *D*_NaBrO_3__ = 1.405 × 10^–5^ cm^2^/s.^48,49^

The propagation of the oxidation waves is no longer localized along the initial contact line, but their nucleation and propagation occur preferentially along this line or along the DLC patterns and mostly in the upper part of the Hele-Shaw cell. At longer times, waves ripple when crossing the DD pattern (see the bottom row of Figure 8).

#### DD Contribution

**Figure 9:**
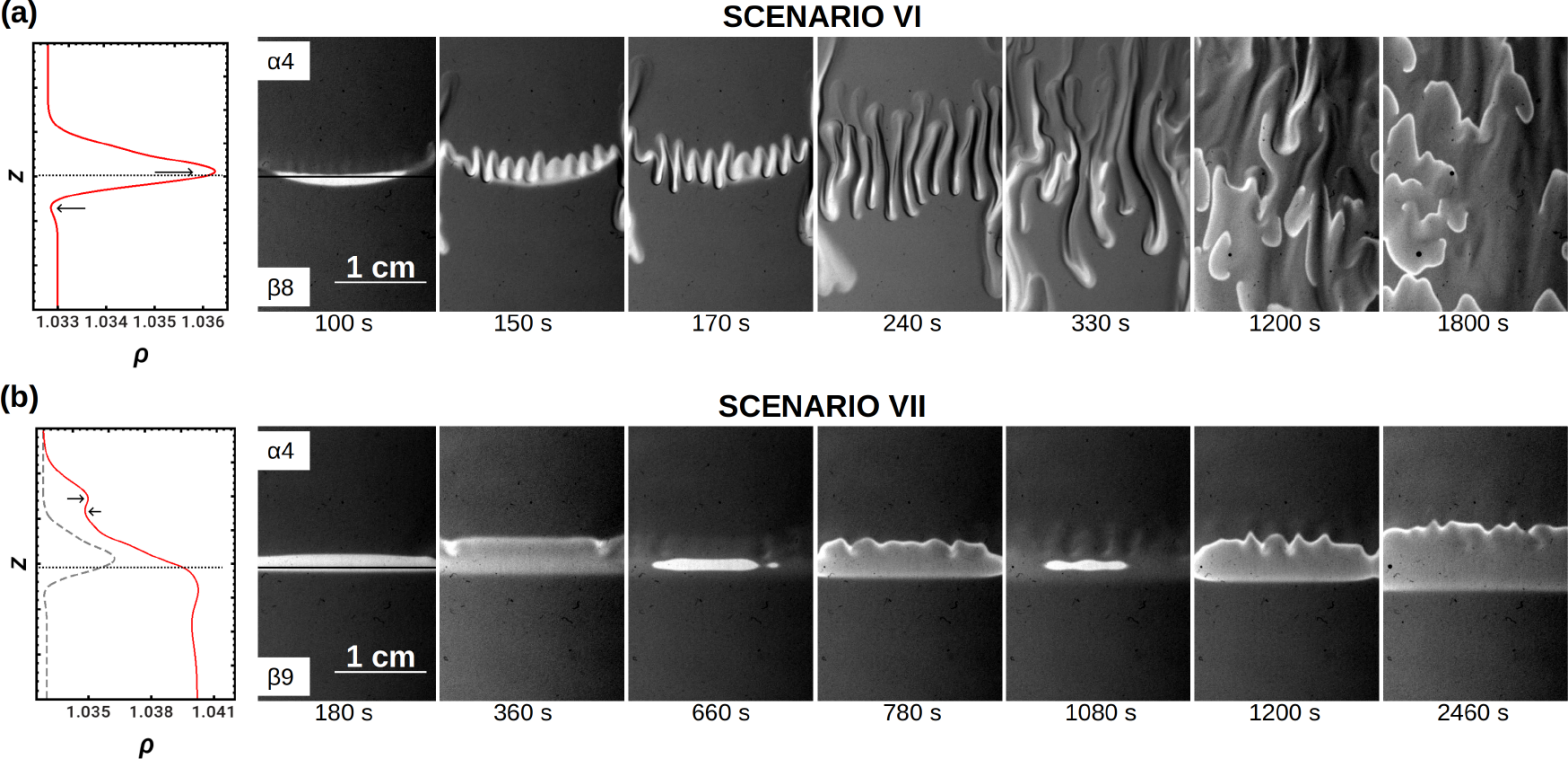
(a) Scenario VI: dynamics observed for the isopycnal case when solution α4 ([BrO_3_^–^] = 0.3 M) is on top of solution β8 ([MA] = 0.6 M). A double-diffusion instability is responsible for the mixing of the reactants in the whole spatial domain. Waves propagate preferentially upward and are broken in segments which are correlated with the wavelength of the convective patterns after these have merged. (b) Scenario VII: deformation of ascending fronts when [MA] = 0.8 M (α4 on top of β9). Note that the stratification is initially statically buoyantly stable. As can be observed from the comparison between the density profile of the isopycnal case (in gray), the stabilization is due to a larger density gap between the top and the bottom solutions. Real size = 2 cm × 3 cm. The horizontal lines in the first snapshots of the dynamics indicate the position of the initial contact line.

When the solutions are reversed, i.e., solution α is now on top (see Figure 9a), double diffusion takes place and dominates the other possible mechanisms.

Scenario VI. For [NaBrO_3_] = 0.3 M and [MA] = 0.6 M, the densities of both solutions are to some degree the same (see Figure 9a). DD fingers deform the contact zone in less than 3 min, before any RD pattern is observed. Later on, waves appear and travel through the whole reactor, preferentially upward in the vertical direction, featuring a rippled and segmented wave pattern^17^ controlled by the wavelength of the hydrodynamic instability.

Scenario VII. Upon increasing [MA] to 0.8 M, i.e., increasing the density difference between the two solutions, another type of dynamics is found (see Figure 9b). Oxidized fronts nucleate along the initial contact line and spread up and down. We thus observe pairs of ascending and descending fronts. Each new front reaches deeper into layers α and β before fading out. Moreover, the rising front ripples, most probably because of a fingering instability which cannot be clearly distinguished. This pattern is reminiscent of the deformation of ascending BZ fronts observed experimentally by Kuhnert et al.^54^ in a reaction mixture open to air. Here the stratification of the reactants gives two possible scenarios of instability: (1) double diffusion due to the less dense bromate salt overlying the denser fast-diffusing sulfuric acid and (2) diffusive layer convection involving sodium bromate and the underlying malonic acid. However, in the previous cases, such instabilities were clearly visible and set in shortly after contact. Moreover only the ascending front is deformed while the descending front does not undergo such periodic deformations. Furthermore, the density difference between the top and bottom solutions is larger for the present case than for the previous cases, which should hinder or delay the onset of either DD- or DLC-type instabilities (see Table 3).

**Table 3:** Summary of the Dynamics Observed Throughout the Experiments^a^

top	bottom	*Δρ*=ρ^bott^ – ρ^top^ (g/cm^3^)	*R=ρ*^bott^/*ρ*^top^	δ=*D*^bott^/*D*^top^	[BrO_3_^–^] M	[MA] M	scenario	figure
β1	α4	0.022	1.0217	0.58	0.30^b^	0	I	4a
β2–5	α4	0.022	1.0216	0.58	0.30^b^	[1 × 10^–3^–1 × 10^–2^]	I	4a
β3	α3	0.010	1.0097	0.58	0.20^b^	2.2 × 10^–2^	I	4a
β3	α2	0.004	1.0043	0.58	0.15^b^	2.2 × 10^–2^	II	4b
α1	β3	0.001	1.0013	1.72	0.10^b^	2.2 × 10^–2^	III	6
β7	α4	0.008	1.0081	0.58	0.30	0.40^b^	IV	8a
β8	α4	≤0.001	1.0002	0.58	0.30	0.60^b^	V	8b
α4	β8	≤0.001	0.9998	1.72	0.30	0.60^b^	VI	9a
α4	β9	0.007	1.0069	1.72	0.30	0.80^b^	VII	9b
β9	α4	–0.007	0.9932	0.58	0.30	0.80^b^	VIII	10a
α4	β7	–0.008	0.9921	1.72	0.30	0.80^b^	IX	10b

a The two first columns give the composition of the two layers placed in contact as detailed in Table 1. The initial difference in density is given with a precision of 0.001 g/cm^3^ in the third column. The fourth column reports the density ratio *R* between the bottom and top solutions. The fifth column shows the diffusivity ratio δ between the bottom and top solutions, essentially controlled by the bromate and sulfuric acid diffusivities. The sixth and the seventh columns further specify the concentrations of bromate and MA, used as control parameters to vary the density jump and the excitability. The last two columns refer to the corresponding scenarios and figures in the text.

b Indicates excitable regimes for scenarios I - III and oscillatory regimes for scenarios IV - IX, as controlled by [BrO_3_^–^] M and [MA] M.

A reaction intermediate could thus be considered to be at the origin of this instability. Bromide ions, which diffuse faster than the bromate ions, *D*_Br^–^_ = 2.08 × 10^–5^ cm^2^/s > *D*_BrO_3_^–^_ = 1.48 × 10^–5^ cm^2^/s,^49^ could start a local double-diffusive mechanism. However, the concentration of this intermediate is on the order of 10^–5^ M and is unlikely to impact the local density profile. Most probably the density profile around the ascending front can be deformed by the local accumulation of denser ferriin coming from the upwardly propagating wave (see the density profile in Figure 9b). This could create a local chemically driven RT instability.

#### Interplay between Oscillatory Dynamics and a Rayleigh–Taylor Instability

**Figure 10:**
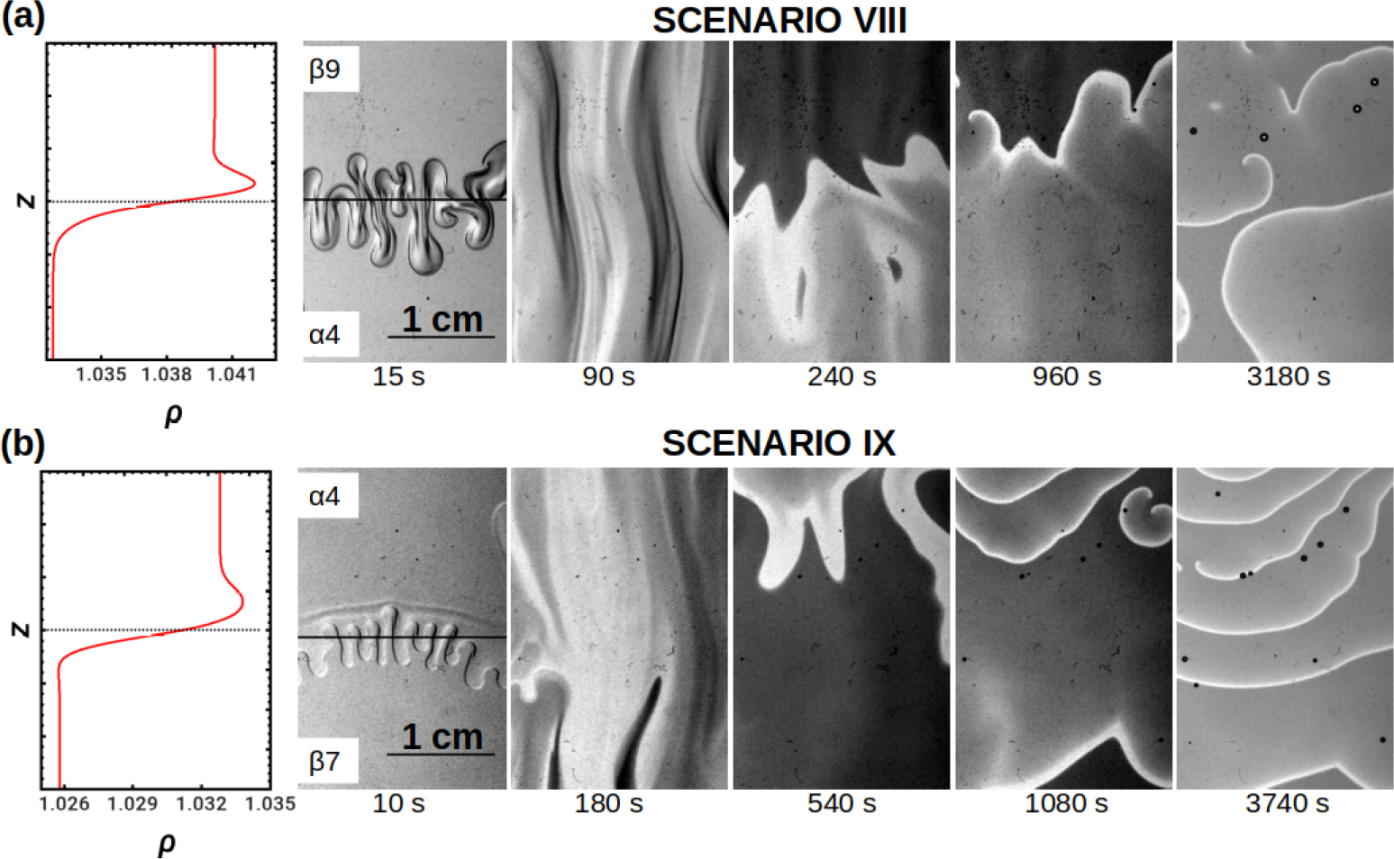
Interplay of localized oscillatory kinetics with an initially unstable stratification. (a) Scenario VIII: propagation of ascending periodic fronts in an oscillatory medium mixed by a Rayleigh–Taylor instability (solution β9, [MA] = 0.8 M, on top of solution α4). Broken waves curl into the beginning of a spiral and further collide with and annihilate the surrounding waves. (b) Scenario IX: propagation of descending fronts after a Rayleigh–Taylor instability causes vigorous stirring through the whole spatial domain (solution β7, [MA] = 0.4 M, underlying solution α4). Real size = 2 cm × 3 cm. The horizontal lines in the first snapshots of the dynamics indicate the position of the initial contact line.

Scenarios VIII and IX. For completeness, we have finally studied the interplay between a Rayleigh–Taylor instability and the oscillating kinetics. We considered both configurations (β over α and *vice versa*) with the top solution denser than the bottom one (*R* < 1) and the concentrations of the initial reactants compatible with an oscillatory regime at the interface ([MA] ∈ [0.4, 0.8] M). The RT mechanism dominates the differential-diffusion-driven instabilities. Figure 10a,b shows the immediate onset of the Rayleigh–Taylor instability after a few seconds, followed by bulk oscillation through the whole spatial domain due to the vigorous convective stirring. Once the catalyst has returned to the reduced state, waves travel preferentially along the vertical direction set up by the vertical convective fingers from side α to side β. The convective motions are not strong enough to completely homogenize the concentration of the main reactants, and the waves can feature rippling and deformation due to residual flow. Moreover, the broken end of a wave can curl like the start of a spiral, though no stable spirals were observed.

Waves appear to be slightly faster than in the initially buoyantly stable counterparts. This effect is attributed to the strong mixing induced by the Rayleigh–Taylor instability which brings fresh reactants into contact in a short time.

## Conclusions

In this work, we have presented a variety of chemohydrodynamic scenarios obtained by localizing the BZ nonlinear kinetics in a double-layer stratification of two solutions containing separated reactants of the BZ system and interacting with classical buoyancy-driven instabilities. (See the summary in Table 3.)

We have investigated systematically the effect of the convective patterns on the reaction–diffusion patterns (and *vice versa*) by activating different types of buoyancy-driven instabilities in combination with both excitable and oscillatory regimes of the chemical system at the initial contact line of the reactant solutions. The type of hydrodynamic instability was selected by considering two initial stratifications of the reactant solution, either malonic acid and sulfuric acid over bromate or *vice versa*. The differential-diffusive interplay between fast-diffusing sulfuric acid and slow-diffusing bromate mainly controls the background buoyancy-driven instability, with the morphology and intensity modulated by changing the density jump between the layered solutions via the bromate concentration. In the concentration range considered, this reactant does not affect the chemical regime which was tuned instead by varying the malonic acid concentration.

The resulting dynamics differ both from the pure reaction–diffusion patterns developing in gel systems^36^ and from the classical nonreactive convective structures.^7−9^ The main features of these chemohydrodynamic scenarios can be summarized as follows.

First, while in the RD system up to two different coexisting wave trains were observed in the transition from the excitable to the oscillatory regime, in the presence of convection only one wave train appears.

Depending on the type of hydrodynamic instability activated, the waves can either remain localized around the contact line or propagate through the whole spatial domain. In particular, the DLC instability (scenarios I, II, and IV) is able to preserve the localization and the properties of the RD patterns, thanks to the diffusive boundary layer between the two independent convective zones. By contrast, DD (scenarios III and VI) and RT (scenarios VIII and IX) instabilities suppress this localization, causing stirring of the reactants through the whole spatial domain, and as a consequence, waves can nucleate and develop throughout the Hele-Shaw cell, being distorted and/or broken by the residual flow. Indeed, when the time scale characterizing the onset of the background convective instability is shorter than that of chemical patterns, flow orients the spatiotemporal behavior of oxidation fronts and waves which travel within hydrodynamic structures. In some cases, several sources of hydrodynamic motions can interplay and the situation becomes quite complex (scenario V).

If the hydrodynamic and chemical time scales are comparable, then the nonlinear chemical kinetics can actively couple and modify the hydrodynamic stability of the stratification. The autocatalytic oxidation which immediately occurs at the solutions’ contact line trigger, for instance, a local RT mechanism due to the density difference between ferroin and denser ferriin. As a consequence, the autocatalytic front, which is planar when only diffusion is at play, here deforms into sinking fingers. When the BZ is locally oscillatory, a transient pulsating fingering can be observed (scenario IV).

The difference in density between the oxidized and reduced forms of the catalyst is not sufficient to cause the onset of density fingering around the periodic waves. Although wave trains are accompanied by density variations, in the downwardly increasing density profile under consideration these are not great enough to produce the periodic onset of a buoyancy-driven instability. We found only one possible case where this could happen (scenario VII, [MA] = 0.8 M), but these patterns would benefit from additional characterizations.

Our results feature a further step toward the control of periodic convective motions by an oscillating chemical reaction. In perspective, it would be interesting to further tune the relative density between the two layers without affecting the dynamical regime of the reaction,^43^ i.e., without varying the concentrations of the reactants, as well as coupling with other convective modes. The former task could be achieved, for instance, by adding small amounts of a spectator salt which is not expected to interfere with the BZ reaction, such as Na_2_SO_4_. For the second task, experiments could be carried out in AOT microemulsions. These water-in-oil emulsions can produce a wealth of reaction–diffusion and reaction–diffusion–convection patterns thanks to their properties as compartmentalized media and to the large cross-diffusion coefficients of their components.^55−58^
